# Room temperature all-solid-state lithium batteries based on a soluble organic cage ionic conductor

**DOI:** 10.1038/s41467-022-29743-1

**Published:** 2022-04-19

**Authors:** Jing Li, Jizhen Qi, Feng Jin, Fengrui Zhang, Lei Zheng, Lingfei Tang, Rong Huang, Jingjing Xu, Hongwei Chen, Ming Liu, Yejun Qiu, Andrew I. Cooper, Yanbin Shen, Liwei Chen

**Affiliations:** 1 Shenzhen Engineering Lab of Flexible Transparent Conductive Films, School of Materials Science and Engineering, Harbin Institute of Technology, Shenzhen, 518055 China; 2 i-Lab, CAS Center for Excellence in Nanoscience, Suzhou Institute of Nano-Tech and Nano-Bionics (SINANO), Chinese Academy of Sciences, Suzhou, 215123 China; 3 School of Materials Science and Engineering, Suzhou University of Science and Technology, Suzhou, 215009 China; 4 Vacuum Interconnected Nanotech Workstation (Nano-X), Suzhou Institute of Nano-Tech and Nano-Bionics, Chinese Academy of Sciences, Suzhou, 215123 China; 5 College of Materials Science and Engineering, Huaqiao University, Xiamen, 361021 China; 6 Leverhulme Centre for Functional Materials Design, Materials Innovation Factory and Department of Chemistry, University of Liverpool, Liverpool, L697ZD, UK; 7 In-situ Center for Physical Sciences, School of Chemistry and Chemical Engineering, Shanghai Jiaotong University, Shanghai, 200240 China

**Keywords:** Nanoscale materials, Batteries, Batteries

## Abstract

All solid-state lithium batteries (SSLBs) are poised to have higher energy density and better safety than current liquid-based Li-ion batteries, but a central requirement is effective ionic conduction pathways throughout the entire cell. Here we develop a catholyte based on an emerging class of porous materials, porous organic cages (POCs). A key feature of these Li^+^ conducting POCs is their solution-processibility. They can be dissolved in a cathode slurry, which allows the fabrication of solid-state cathodes using the conventional slurry coating method. These Li^+^ conducting cages recrystallize and grow on the surface of the cathode particles during the coating process and are therefore dispersed uniformly in the slurry-coated cathodes to form a highly effective ion-conducting network. This catholyte is shown to be compatible with cathode active materials such as LiFePO_4_, LiCoO_2_ and LiNi_0.5_Co_0.2_Mn_0.3_O_2_, and results in SSLBs with decent electrochemical performance at room temperature.

## Abstract (web summary)

While solid-state batteries offer higher energy densities than liquid-based batteries, such devices require effective ion conduction pathways. Here, authors prepare porous organic cages as solution-processable catholytes that are enable excellent performances from various cathode active materials.

## Introduction

Solid-state lithium (Li) batteries have theoretically higher energy densities and better safety characteristics than organic solvent-based Li-ion batteries^1,2^. Research in the solid-state battery field has focused mostly on developing solid-state electrolytes (SSEs)^3–6^ and improving their interfaces with the cathode and anode^7–9^. This has yielded various promising SSEs with bulk Li^+^ ion conductivity in the 10^−6^–10^−3^ S cm^−1^ range and various technical solutions have been developed to address the solid electrode/electrolyte interfacial problem^10–13^. A critical but less addressed problem, however, lies in the Li^+^ ion transport in solid-state cathodes, especially when thick cathodes with practically meaningful areal loadings are considered^14,15^.

So far, there are three main strategies to construct the Li^+^ transport networks inside solid-state cathodes. The first approach is to add a defined amount of liquid electrolyte, ionic liquid, or plastic crystal with a Li salt to a porous cathode structure^16–19^; for example, 50~100 µL plastic crystal electrolyte^16^ or 20% succinonitrile/LiTFSI^19^ were added to solid-state cathodes to obtain good ionic conductivity. However, for high energy density batteries with high capacity and high voltage electrode materials, such as metallic Li anode, Ni-rich layered cathode, and lithium nickel manganese spinel cathodes, most of the commonly used organic electrolytes tend to decompose due to their narrow electrochemical windows and form SEI or CEI, causing the cell capacity to “rollover” when the liquid in the electrode is depleted after repeated cycles. As such, removing all liquid components to realize all-solid-state batteries with high safety and high energy density is the ultimate goal. A second approach is to introduce inorganic SSEs as catholytes, such as Li_7_La_3_Zr_2_O_12_ (LLZO) and Li_10_GeP_2_S_12_ (LGPS), in solid-state cathodes^20,21^. Inorganic SSEs are insoluble and can only be added in the form of particles, which are prone to aggregation and poor dispersion in the cathodes. Hence, to ensure effective ion transport in the cathodes, a large amount of the inorganic SSEs is often needed in the cathode mixture, typically 20–60 wt%, which inevitably lowers the energy density. Moreover, poor solid-solid contact between the ceramic SSE particles and active material can result in high energy barrier for Li ion transport^22,23^. A third approach is to add polymer electrolytes with Li salts to facilitate ion transport. However, most polymer electrolytes still face problems of having a narrow electrochemical window and low ion conductivity at room temperature^24–26^. As a result of these various limitations, few reports on room temperature SSLB have displayed satisfactory electrochemical performance for future practical applications^27^. For example, high amounts (30–60%) of SSE are often needed in solid-state cathodes^20,21,28^, and complicated preparation procedures may be required^29^ to construct solid-state cathodes, sometimes resulting in only limited cycle life^30^. As such, the construction of efficient Li^+^ transport networks in stable solid-state cathodes are important and urgent if we are to build SSLBs with satisfactory performance at room temperature.

Here we report the use of a soluble organic cage-based Li^+^ conductor as catholyte for room temperature SSLBs. Porous solids, such as metal–organic frameworks (MOFs)^31,32^ and covalent organic frameworks (COFs)^33–35^, have been explored extensively for their ion conduction properties. Unlike MOFs and COFs, which are extended, insoluble frameworks, organic cages have discrete molecular covalent structures, and can be solution processable^36^. Such molecules can pack together to form crystals with highly interconnected three-dimensional pore networks. The discrete nature of cage molecules renders them soluble in different solvents^37^, which offers processability and can introduce different physical properties by mixing with other soluble compounds^38–40^. For example, a series of crystalline porous amine cages was developed for proton conduction, and the resulting proton conductivities were comparable with MOFs^41^. However, to date, there have been no Li ion conductors based on porous organic cages.

In the organic cage-based solid-state Li^+^ conductor developed in this work, ionized functional groups in the organic cage framework provide an environment with high effective dielectric screening, thus allowing an added Li salt, such as LiClO_4_, to dissociate into mobile ions. In addition to exhibiting highly desirable room temperature ionic conductivity, a key feature of this organic cage-based ion conductor is its solubility in polar solvents. Hence, the organic cage Li^+^ conductor is readily incorporated into solid-state cathodes as a catholyte in the slurry mixing step. The cage catholyte, which is dissolved uniformly in the cathode slurry, crystallizes upon solvent evaporation and grows on the surface of the cathode particles during the coating process, thus forming an effective ion conduction network inside the cathode. This approach of using organic cages in SSLBs can minimize the quantity of ionic additive needed for the cathode and leads to excellent room temperature cycling performance.

## Results and discussion

### Synthesis and characterization of Li-RCC1-ClO_4_

**Fig. 1:**
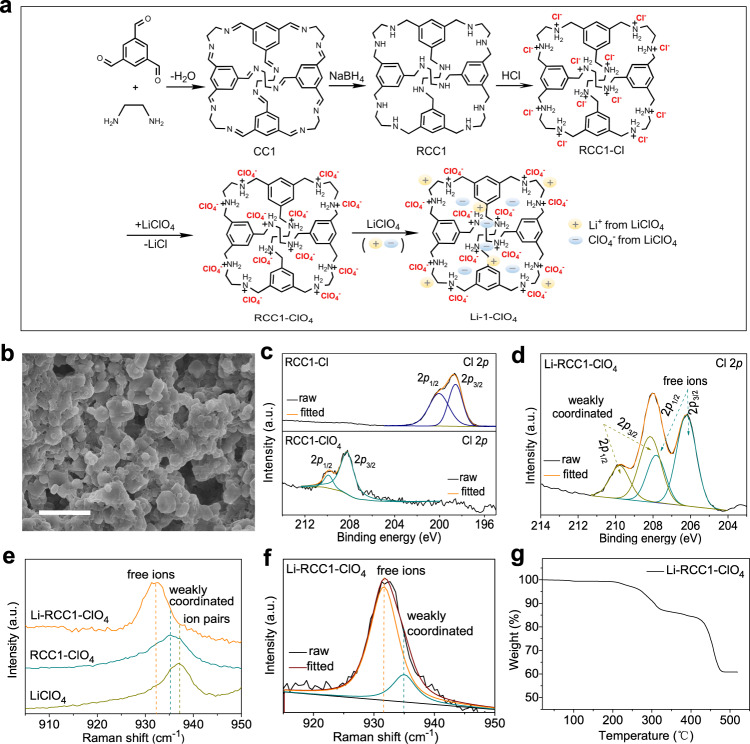
Synthesis and characterization of the cage-based SSE, Li-RCC1-ClO_4_. **a** Synthetic procedure for the porous cage electrolyte Li-RCC1-ClO_4_. Note: the light yellow and blue symbol represent Li ion and ClO_4_ ion from LiClO_4_, respectively. **b** SEM micrograph of Li-RCC1-ClO_4_ powder. **c**, **d** XPS Cl 2*p* spectra of RCC1-Cl, RCC1-ClO_4_, and Li-RCC1-ClO_4._ **e** Raman spectra of LiClO_4_, RCC1-ClO_4_ and Li-RCC1-ClO_4_. **f** Fitting of the Li-RCC1-ClO_4_ Raman spectrum in the range of 915~950 cm^−1^. **g** Thermogravimetric analysis for Li-RCC1-ClO_4_ under N_2_ atmosphere (heating rate: 10 °C/min)_._ Scale bar for **b** is 5 μm.

As shown in Fig. 1a, the SSE was derived from a three-dimensional porous organic cage, RCC1-Cl, which comprises of a cationic amine cage framework (RCC1) and chloride counterions, as used previously as a proton conductor^41,42^. Here, the chloride counterions in RCC1-Cl were exchanged with perchlorate ions, resulting in a different cage, RCC1-ClO_4_, which was then blended with LiClO_4_ salt to form a SSE, Li-RCC1-ClO_4_.

Scanning electron microscope (SEM) images (Supplementary Fig. 1), X-ray diffraction (XRD) patterns (Supplementary Fig. 2) and nuclear magnetic resonance (NMR) spectra (Supplementary Fig. 3) collected for the RCC1-Cl sample are consistent with previous reports^42^. The SEM image in Fig. 1b shows that sub-micron-sized particles of the Li-RCC1-ClO_4_ material crystallize from an ethanol/water solvent mixture (v:v = 3:1) and these are interconnected with each other to form a fluffy aggregate. The energy-dispersive X-ray spectroscopy (EDX) measurement of the prepared Li-RCC1-ClO_4_ solid pellet presented in Supplementary Fig. 4 shows that the LiClO_4_ is dispersed uniformly in the solid. X-ray photoelectron spectroscopy (XPS) was used to monitor the formation of the Li-RCC1-ClO_4_ SSE. As shown in Fig. 1c, the RCC1-Cl sample shows the pair of characteristic Cl 2*p* peaks at 200.0 and 198.4 eV, indicating the bonding of the chloride ions to the RCC1 cage^43,44^; while the Cl 2*p* peaks obtained from the RCC1-ClO_4_ sample appears at 209.7 and 208.2 eV, which is slightly lower in binding energy than that of LiClO_4_ salt (211.0 and 209.4 eV)^45^, indicating that the chloride ions have been exchanged to perchlorate ions and the perchlorate ions are weakly coordinated to the -NH_2_^+^- groups on the cage skeleton. After adding LiClO_4_ salt to the RCC1-ClO_4_, two distinct sets of Cl 2*p* peaks can be observed. One set of the Cl 2*p* peaks is found at 209.8 eV and 208.3 eV (Fig. 1d), which is similar to peaks observed for weakly-coordinated perchlorate ions (Fig. 1c); another set of Cl 2*p* peaks appears at lower binding energies of 207.8 eV and 206.2 eV, which might be attributed to free perchlorate ions that dissociated from the added LiClO_4_ salt (the molar ratio of [Li^+^]/[-NH_2_^+^-] is ~1:1; the influence of the salt concentration on the Li-RCC1-ClO_4_ SSE is discussed in later sections).

The existence of perchlorate ions in different chemical environments was also observed in Raman spectra. As shown in Fig. 1e, the LiClO_4_ reference sample shows a characteristic sharp peak at ~937 cm^−1^, which can be assigned to the Li^+^/ClO_4_^−^ ion pairs, while both the RCC1-ClO_4_ and Li-RCC1-ClO_4_ samples exhibit characteristic peaks at a lower wavelength (~935 cm^−1^ and ~932 cm^−1^, respectively) that can be assigned for weakly coordinated and free perchlorate ions^46–48^. Based on the integrated peak areas, it can be estimated that the free and weakly coordinated perchlorate ions in the Li-RCC1-ClO_4_ SSE are about 82% and 18%, respectively (Fig. 1f), assuming that the extinction coefficients for the Raman peaks are comparable. The perchlorate ions in the Li-RCC1-ClO_4_ SSE are therefore either weakly coordinated to the RCC1 cage skeleton or in a “free” form; this suggests that the Li^+^ in the LiClO_4_ salt should be well dissociated from the anions, which is beneficial for ionic conductivity. The Li-RCC1-ClO_4_ SSE also has a good thermal stability: it only starts decomposing at temperatures higher than 220 °C (Fig. 1g). By contrast, many polymer electrolytes, such as PEG-based materials, will decompose at temperature below 200 °C^49,50^.

### Electrochemical performance of Li-RCC1-ClO_4_

**Fig. 2:**
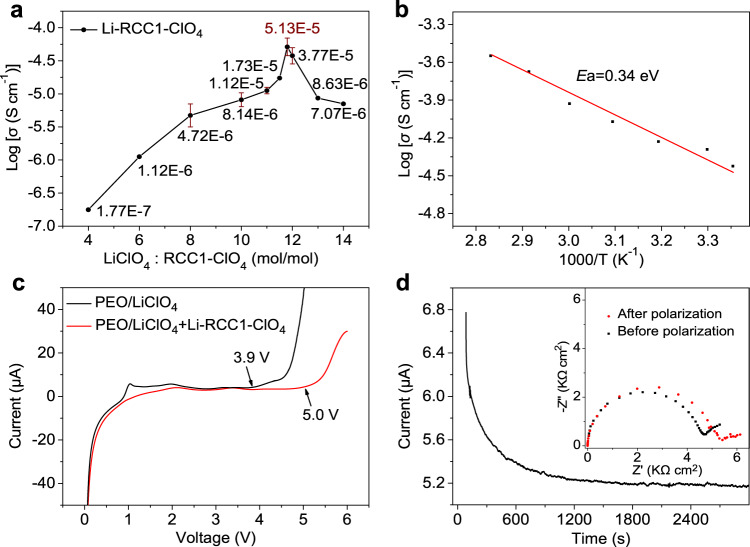
Electrochemical characterization of Li-RCC1-ClO_4._ **a** Ion conductivity of the Li-RCC1-ClO_4_ SSE with different LiClO_4_ contents. **b** Ion conductivity of Li-RCC1-ClO_4_ as a function of temperature. **c** Linear sweep voltammetry (LSV) results of PEO/LiClO_4_ and PEO/Li-RCC1-ClO_4_ at a scan rate of 1 mV s^−1^. **d** Time-dependent current before and after direct-current (DC) polarization (with a DC voltage of 50 mV). The insert image shows the EIS results of Li-RCC1-ClO_4_ symmetric cell before and after polarization.

The ionic conductivity of the Li-RCC1-ClO_4_ SSE is sensitive to the molar ratio of [Li^+^]/[-NH_2_^+^-]. As calculated based on the resistance obtained from electrochemical impedance spectroscopy (EIS) measurements (Fig. 2a and Supplementary Fig. 5), the room temperature ion conductivity of the Li-RCC1-ClO_4_ SSE increases as the content of the LiClO_4_ increases, and reaches a peak value of 5.13 × 10^−5^ S cm^−1^ at a molar ratio [Li^+^]/[-NH_2_^+^-] of about 1. Further increase in the content of the Li salt to a molar ratio of [Li^+^]/[-NH_2_^+^-] greater than 1:1 will result in an increase in residual undissociated LiClO_4_ (Supplementary Fig. 6), leading to decreased ion conductivity. Supplementary Fig. 7 and Fig. 2b show the ionic conductivity of the Li-RCC1-ClO_4_ SSE at different temperatures, which reaches 1.2 × 10^−4^ S cm^−1^ at 60 °C; the activation energy is calculated to be 0.34 eV. This room temperature conductivity is considerably higher than some polymer SSEs, such as PEO and PVDF SSEs^51,52^. More importantly, the Li-RCC1-ClO_4_ SSE show a wide electrochemical window up to 5.0 V (Fig. 2c) and a very high ion transference number of ~0.7 is obtained for the Li-RCC1-ClO_4_ SSE (Fig. 2d and Supplementary Fig. 8), confirming the disassociation of LiClO_4_ and suggesting that the -NH_2_^+^- groups in the cage molecule restrict movement of the ClO_4_^−^ anions.

This is the first report of a porous organic material that can be used as Li ion solid-state electrolyte without any solvent. It has a room temperature ionic conductivity of 5.13 × 10^−5^ S cm^−1^, an electrochemical window up to 5.0 V, and a transfer number of ~0.7. More importantly, the discrete molecular structure of the cage makes it soluble in common polar solvents, and thus can be facilely mixed with electrode materials by slurry-coating, an industrial compatible electrode preparation process. As such, this cage-containing electrolyte is advantageous for constructing ionic conducting pathways inside solid-state cathodes.

### Application of Li-RCC1-ClO_4_ in all-solid-state cathodes

**Fig. 3:**
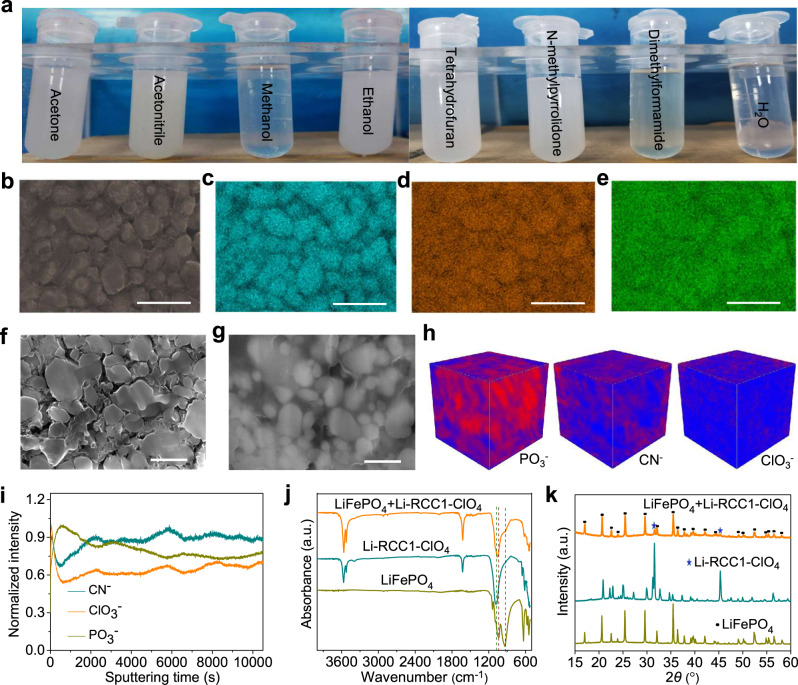
Characterization of Li-RCC1-ClO_4_ in all-solid-state cathodes. **a** Solubility of Li-RCC1-ClO_4_ in different solvents. **b** SEM image and **c**–**e** EDS elemental mappings of Fe, N and Cl in the cathode with Li-RCC1-ClO_4_. SEM micrographs of focus-ion beam cut cross-section of the solid cathodes without (**f**) and with Li-RCC1-ClO_4_ (**g**). **h** TOF-SIMS 3D renderings of the solid cathode with Li-RCC1-ClO_4_. **i** Normalized TOF-SIMS depth profiles of the solid cathode with Li-RCC1-ClO_4_. **j** FTIR spectra of LiFePO_4_, Li-RCC1-LiClO_4_ and mixture of LiFePO_4_ and Li-RCC1-ClO_4_. **k** XRD patterns of LiFePO_4_, Li-RCC1-LiClO_4_ and mixture of LiFePO_4_ and Li-RCC1-ClO_4_. Scale bars for **b**–**g** are all 500 nm.

Li-RCC1-ClO_4_ has potential as an SSE because it exhibits an ionic conductivity of 5.13 × 10^−5^ S cm^−1^ at room temperature, which is comparable to the best-performing polymer SSEs reported^53,54^. It is particularly promising for all-solid-state cathodes because of its processing advantages. Unlike insoluble network polymers and extended frameworks, such as MOFs and COFs, this porous organic cage can be dissolved in a variety of solvents, such as water and methanol (10 mg mL^−1^, Fig. 3a), offering a range of solution-processing options. Here, the ion-conducting organic cage Li-RCC1-ClO_4_ was used to address the ion conduction issue in solid-state cathodes. The solution processibility of the organic cage allows the problem to be tackled with the conventional slurry coating method, which is the process used in the manufacturing of current liquid electrolyte batteries. The solid-state cathode contained LiFePO_4_ as the active material, acetylene black (AB) and carbon nanotubes (CNTs) as electronic conductors, polyvinylidene fluoride (PVDF) as a binder, and Li-RCC1-ClO_4_ as the ionic conductor, combined in the weight ratio LiFePO_4_:AB:CNTs:PVDF:Li-RCC1-ClO_4_ = 71:4:2:3:20 (that is, 20 wt.% of the organic SSE). Methanol/N-methylpyrrolidone (NMP) was used as the solvent (Supplementary Fig. 9).

As shown in Supplementary Fig. 10 and Fig. 3b, the LiFePO_4_ particles in the resulting solid-state cathode are interconnected by a binder-like substance, which was identified by elemental mapping of EDX to be the Li-RCC1-ClO_4_ SSE. As shown in Fig. 3c–e, the Fe signal from LiFePO_4_ shows a clear particle shape (Fig. 3c); on the other hand, the N and Cl signals arise from the Li-RCC1-ClO_4_ (Fig. 3d, e) are distributed throughout the field of view, surrounding these LiFePO_4_ particles. These observations suggest that Li-RCC1-ClO_4_ crystallizes from the NMP/methyl alcohol mixture and forms an ionic conducting network surrounding and interconnecting the LiFePO_4_ particles. In sharp contrast, a cathode prepared using the same method but without Li-RCC1-ClO_4_ was discontinuous and most of the LiFePO_4_ particles were isolated from each other (Supplementary Fig. 11a–c). EDX mapping for this cathode (Supplementary Fig. 11d–h) also showed a clear particle-shaped Fe signal but negligible signals from N and Cl. Similar results were observed in the depth direction of the cathode using cross sectioned samples prepared by focus-ion-beam (FIB) milling. As shown in Fig. 3f, g, SEM images for cathode cross sections show that most LiFePO_4_ particles are isolated from each other without the Li-RCC1-ClO_4_ additive (Fig. 3f), while the LiFePO_4_ particles in the cathode with the Li-RCC1-ClO_4_ SSE are interconnected by the ion conductor (Fig. 3g).

Time-of-flight secondary ion mass spectrometry (TOF-SIMS) was also used to assay the distribution of the catholytes inside the cathode. Figure 3h, i show the 3D chemical images and depth profiling of the cathode with Li-RCC1-ClO_4_, in which the PO_3_^−^, CN^−^ (and ClO_3_^−^) fragments can be attributed to the LiFePO_4_ and Li-RCC1-ClO_4_, respectively. As shown in Fig. 3h, the PO_3_^−^ fragments occupy most of measured volume, while the CN^−^ and ClO_3_^−^ fragments fill the remaining space. The corresponding normalized depth profile in Fig. 3i also reveals that the distribution of the PO_3_^−^, CN^−^ (and ClO_3_^−^) fragments through the cathode are complementary, confirming that the LiFePO_4_ particles are surrounded homogeneously by the Li-RCC1-ClO_4_ catholyte.

Fourier transform infrared (FTIR) spectra (Fig. 3j) showed that the characteristic absorption peaks of Li-RCC1-ClO_4_ SSE are unchanged in the LiFePO_4_ solid-state cathode, indicating stability of the cage catholyte. The powder X-ray diffraction (PXRD) pattern (Fig. 3k) shows that the characteristic diffraction peak (marked with asterisk) of Li-RCC1-ClO_4_ is retained, suggests that the crystal structure of Li-RCC1-ClO_4_ is also preserved. However, there is an obvious loss of crystallinity in the slurry-coated solid cathode in comparison to Li-RCC1-ClO_4_ in its pure powder form. We speculate that the interactions between LiFePO_4_ and the Li-RCC1-ClO_4_ SSE, likely the ionic interactions between NH_2_^+^ on cages and PO_4_^−^ from LiFePO_4_, may affect the recrystallization of the cage in slurry coating and also allow Li-RCC1-ClO_4_ to grow into a thin layer on the surface of the LiFePO_4_ particles, rather than crystallize into a large crystalline particle during the slurry coating process; this could in turn affect the relative intensity of the PXRD peaks due to preferred orientation. This morphology change, however, also results in more efficient utilization of the cage catholyte with respect to insoluble particulate SSEs, and we believe that helps to create continuous ionic conducting pathways and to reduce interfacial resistance by using a relatively small dose of the additive.

### Electrochemical performance of all-solid-state batteries with Li-RCC1-ClO_4_ as catholyte

**Fig. 4:**
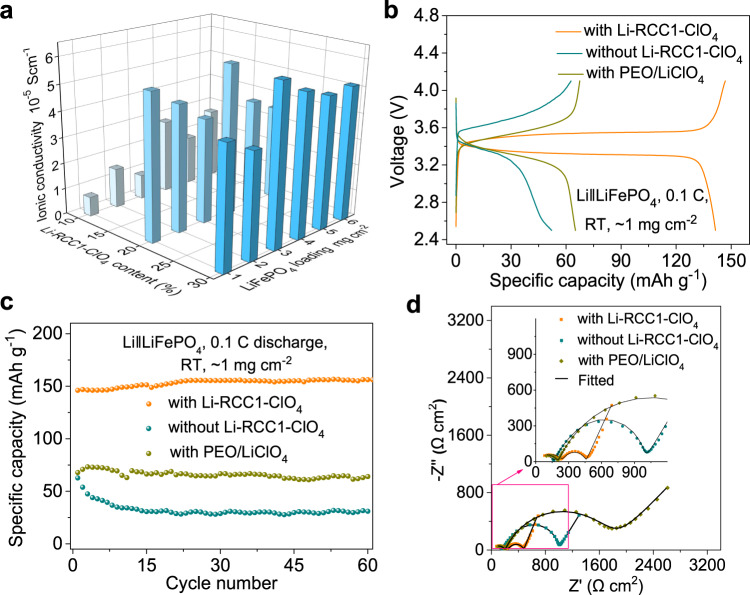
Electrochemical performance of the all-solid-state battery with ionic conducting cage catholyte Li-RCC1-ClO_4_. **a** Comparison of the ionic conductivities of the solid cathodes with different Li-RCC1-ClO_4_ content and LiFePO_4_ areal loading. Note: the cyan from light to dark symbol represent solid-state cathode with 10%, 20% and 30% Li-RCC1-ClO_4_ electrolyte, respectively; The detailed values are provided in Supplementary Table 1. **b** Voltage profiles, **c** cycling performance, and **d** Electrochemical impedance spectra profiles of the SSLBs at room temperature using LiFePO_4_ solid-state cathodes (loading: ~1.0 mg cm^−2^) with and without Li-RCC1-ClO_4_, as well as with PEO/LiClO_4_ polymer as the catholyte. The inset in 4d shows the enlarged impedance curves of the red square. The fitted modal is shown in Supplementary Fig. 15.

The Li-RCC1-ClO_4_ catholyte in the LiFePO_4_ solid-state cathode was optimized by comparing the specific capacity and polarization of the SSLB. For solid-state cathodes with 0, 10, 20 and 30 wt% Li-RCC1-ClO_4_ additive (Supplementary Fig. 12), the specific capacity increases and the polarization decreases as the Li-RCC1-ClO_4_ content increases from 0 wt% to 20 wt%. The performance then deteriorates when the amount of Li-RCC1-ClO_4_ is increased further to 30 wt%. Higher Li-RCC1-ClO_4_ contents results in increased polarization, probably because the electronic conducting pathway is negatively affected by the ionic conducting additive. The optimized additive content was therefore set to be 20 wt%, which is significantly less than the more typical values of 30–60 wt% for inorganic SSE additives^28,29,55^. Furthermore, the ionic conductivities of the solid cathodes with different Li-RCC1-ClO_4_ content and LiFePO_4_ areal loading were investigated systematically. As shown in Fig. 4a and Supplementary Table 1, when the content of Li-RCC1-ClO_4_ was higher than 20%, the ionic conductivities of solid-state cathodes with 1, 2, 3, 4, 5, 6 mg cm^−2^ of active materials were similar, indicating that Li-RCC1-ClO_4_ could construct effective ionic conductivity network in thick solid-state cathode. The average ionic conductivity of the cathode with 20% and 30% Li-RCC1-ClO_4_ was 4.64 × 10^−5^ and 5.06 × 10^−5 ^S cm^−1^, respectively. Even though the latter has a slightly higher conductivity, 20% of Li-RCC1-ClO_4_ catholyte was still the best choice when taking energy density and electronic conductivity into consideration (too much electrolyte might hinder the electron conduction).

The LiFePO_4_ solid-state cathode (with a weight ratio of LiFePO_4_:AB:CNTs:PVDF: Li-RCC1-ClO_4_ = 71:4:2:3:20) was then assembled with a lithium foil anode and a polymer solid electrolyte, P(IL-PEGDA), which was dried and stored in glove box for one week to remove residual solvent, as reported in our previous work^56^, to form a coin cell SSLB. This could be used to light an LED (Supplementary Fig. 13). Figure 4b, c compares the electrochemical performance of coin cells using LiFePO_4_ solid-state cathodes (loading: ~1.0 mg cm^−2^) with and without the Li-RCC1-ClO_4_ catholyte, as measured at room temperature. To compare with commonly used polymer ion conductors, we also evaluated the performance of LiFePO_4_ solid-state cathode containing PEO/LiClO_4_ as the catholyte instead of the Li-RCC1-ClO_4_. As shown in Fig. 4b, the cell with Li-RCC1-ClO_4_ catholyte exhibits discharge and charge profile with a voltage plateau at ~3.4 V and an initial specific capacity of ~147 mAh g^−1^ at 0.1 C, similar to a LiFePO_4_ cell with liquid electrolyte^57^. By contrast, very large polarization and limited initial specific capacity was observed from cells without Li-RCC1-ClO_4_ (~530 mV, 52 mAh g^−1^) or with the PEO/LiClO_4_ catholyte (~700 mV, 65 mAh g^−1^). Without the Li-RCC1-ClO_4_ catholyte, or with the PEO/LiClO_4_ catholyte, much of the LiFePO_4_ material in the cells is unutilized, or underutilized, because of the absence a sufficient ionic conducting network in the solid-state cathodes^58^. Their corresponding cycling performance and coulombic efficiency is shown in Fig. 4c and Supplementary Fig. 14, respectively. The SSLB cells containing the Li-RCC1-ClO_4_ catholyte delivers an initial discharge capacity of 147 mAh g^−1^ and can run stably with capacity of 152 mAh g^−1^ at 0.1 C with stable coulombic efficiency nearly 100%; while the battery without Li-RCC1-ClO_4_ additive or with PEO/LiClO_4_ as catholyte presents extremely low capacity and floating coulombic efficiency almost between 95–100%. These are consistent with EIS results, shown in Fig. 4d, which reveal that the total impedance of the SSLB with the Li-RCC1-ClO_4_ catholyte is ~468 Ω cm^2^; that is, much lower than the 992 Ω cm^2^ measured for the battery without Li-RCC1-ClO_4_ and the 1737 Ω cm^2^ measured for the battery containing PEO/LiClO_4_. The Nyquist plots of the Li‖LiFePO_4_ cells with different catholytes show two semicircles (Fig. 4d). To construct the equivalent circuit for the EIS data, Li|SSE|Li and SS|SSE|SS symmetry cells were assembled and investigated (Supplementary Fig. 15) to help identifying each part of the resistance in the Li‖LiFePO_4_ cell shown in Fig. 4d. As shown in Supplementary Fig. 15a, the semicircle (~100 Ω cm^2^) for the SS|SSE|SS cell at high frequency (>1.5 MHz) is assigned to the resistance of solid-state electrolyte, and the semicircle (~220 Ω cm^2^) for the Li|SSE|Li cell at middle and low frequency (the apex value of the semicircle is 2.2 KHz) is attributed to the Li|SSE interfacial charge transfer resistance. And the semicircle at the low frequency (the apex value of the semicircle is 320 Hz) should be attributed to the cathode|SSE interfacial charge transfer resistance^19,29^. Based on these results, equivalent circuit used for fitting the EIS of three different Li‖LiFePO_4_ cells is obtained (Supplementary Fig. 15b). A summary of the fitting results is given in Supplementary Table 2. In which all of the fitting results of *X*^2^ are close to 10^−3^, indicating a good fitting of the EIS data. With the Li-RCC1-ClO_4_ additive, the cathode|SSE interface showed a relatively low resistance of ~255 Ω cm^2^ at RT, which is much smaller than 785 and 1520 Ω cm^2^ for the cell without Li-RCC1-ClO_4_ and with PEO/LiClO_4_ catholyte, respectively, suggesting the successful construction of effective ion conducting pathway in the cathode by Li-RCC1-ClO_4_ catholyte.

**Fig. 5:**
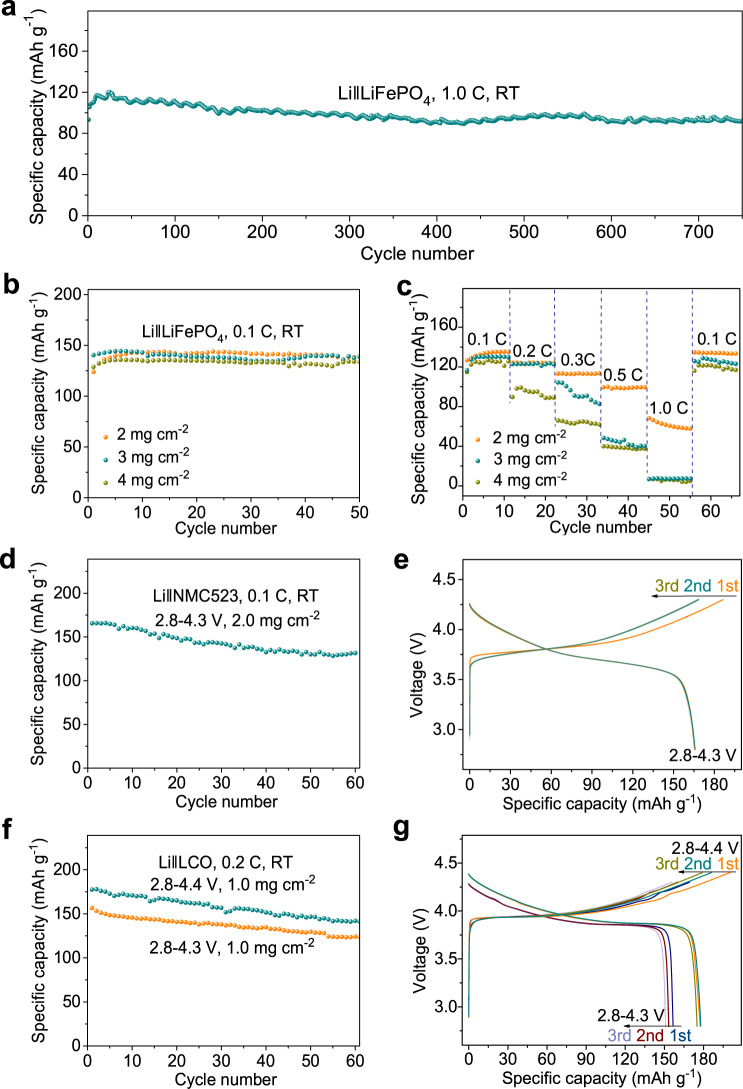
Electrochemical cycling of the all-solid-state battery with Li-RCC1-ClO_4_ catholyte. **a** Cycle performance of the all-solid-state cell with Li-RCC1-ClO_4_ at 1.0 C under room temperature (loading: ~1.0 mg cm^−2^, oscillations are due to variations in the ambient temperature). **b** Cycling performance, and **c** rate performance of the SSLBs at room temperature with different LiFePO_4_ areal loading. **d** Cycle performance and **e** voltage-capacity curves of the all-solid-state cell with NCM523 cathode during 2.8~4.3 V at 0.1 C under room temperature. **f** Cycle performance and **g** voltage-capacity curves of the all-solid-state cell with LCO cathode during 2.8~4.3 V and 2.8~4.4 V at 0.2 C under room temperature.

We also evaluated the LiFePO_4_ solid-state cathode at higher charge/discharge rates and at higher cathode loadings, since these are important parameters that govern the cell power density and energy density of the SSLB. When the current of the 1.0 mg cm^−2^ LiFePO_4_ solid-state cathode was increased to 0.5 C, it showed a specific capacity of 122~135 mAh g^−1^ during 200 cycles at room temperature (Supplementary Fig. 16a). The initial coulombic efficiency of the ASSLMB is 93.4%, which subsequently increases to 99.9% and 100.0% in the second and third cycle, and maintained nearly 100% in the following 200 cycles at 0.5 C (Supplementary Fig. 16b). Its corresponding voltage-capacity curves were shown in Supplementary Fig. 17. This result suggests that the contact between the electrode material and the electrolyte is good during charge and discharge. Further increased the current to 1.0 C, the LiFePO_4_ SSLB shows even better cycle stability, with 88.2% capacity retention at the 750^th^ cycle, corresponding to 0.026% capacity decay per cycle (Fig. 5a and Supplementary Fig. 18). The morphology of the LiFePO_4_ cathode after 750 cycles was investigated by SEM. As shown in Supplementary Fig. 19, there was no obvious changes, such as cracks or deformation, in the cycled LiFePO_4_ cathode when compared with the LiFePO_4_ cathode before cycling (Supplementary Fig. 10), demonstrating the Li-RCC1-ClO_4_ catholyte can maintain the morphology of the solid cathode during charge-discharge processes.

Ion-conduction in thick solid cathode will be more challenging. As shown in Fig. 5b and Supplementary Fig. 20, there were no obvious difference in the 0.1 C cycle life of the all-solid-state batteries with different LiFePO_4_ areal loadings of 2, 3 and 4 mg cm^−2^. However, rate performance of the batteries differed by areal loading (Fig. 5c). The discharge capacity of the battery with a thicker cathode declines faster as the current density increases. This may be attributed to the coarse and fluffy deposited Li at high areal capacity, which result in poor solid-solid contact between the Li metal anode and solid electrolyte^59,60^. Thus, the surface morphology of the lithium anodes before and after cycled at different current densities were further investigated, as shown in Supplementary Fig. 21. The lithium foil presented was observed to become coarse and fluffy as the LiFePO_4_ areal loading increased from 2 to 4 mg cm^−2^, indicating that the lithium anode side is problematic with high areal capacity.

As shown in Supplementary Fig. 22a, when the rate of the 2 mg cm^−2^ LiFePO_4_ solid-state cathode was increased from 0.1  to 0.5 C at room temperature, it still delivered an initial specific capacity of ~90 mAh g^−1^, which slowly increased to ~115 mAh g^−1^ in subsequent cycles. When both the C-rate and temperature were increased at the same time to 0.5 C and 60 °C, the 2 mg cm^−2^ LiFePO_4_ solid-state cathode showed a discharge capacity of ~135 mAh g^−1^ with a relatively stable cycle life, which was close to that obtained at 0.1 C at room temperature (Supplementary Fig. 22b).

The capacity and cycle stability of the cell with the Li-RCC1-ClO_4_ catholyte at 60 °C was much better than that for the PEO/LiClO_4_ catholyte, which exhibited a specific capacity of ~90 mAh g^−1^ and a capacity retention of 75.6% after 50 cycles (Supplementary Fig. 23). Additional cycling results for the Li-RCC1-ClO_4_ containing cells with different cathode loadings are provided in Supplementary Fig. 24. This SSLB performance is among the best reported in the literature (Supplementary Fig. 25 and Supplementary Table 3)^21,61–67^, particularly in terms of the room-temperature performance and long-term cycling performance.

To check whether the polymerized electrolyte layer has any contribution to the ionic conductivity of the solid-state cathode, elemental mappings were made on the cross section of the SSLB containing the Li-RCC1-ClO_4_ additive (cathode loading: ~1.0 mg cm^−2^) after 50 cycles at 0.1 C/room temperature. This was mainly done to see whether the polymerized electrolyte had diffused into the cathode electrode during cycling. As shown in Supplementary Fig. 26, the characteristic elements Al of the Al foil, Fe of the LiFePO_4_ cathode and the S of the polymerized electrolyte are observed on the current collector, the cathode, and the electrolyte layer, respectively, with a clear boundary between each. No characteristic element S of the polymerized electrolyte could be observed in the cathode layer, suggesting that no electrolyte diffused into the cathode during the cycling.

This approach is transferable to other systems: for example, this slurry coating process works not only for polymer SSEs, but is also suitable for ceramic SSEs, such as garnet-type LLZO. As shown in Supplementary Fig. 27, an Li|LLZO|LiFePO_4_ cell with the Li-RCC1-ClO_4_ additive showed a discharge capacity of ~130 mAh g^−1^, while the cell without the additive hardly delivered any capacity.

This Li-RCC1-ClO_4_ catholyte was also applied in SSLBs with high-voltage LiNi_0.5_Co_0.3_Mn_0.3_O_2_ (NCM523) and LiCoO_2_ (LCO) cathodes. The solid state NCM523 cell delivers an initial discharge capacity of 165 mAh g^−1^ and an initial coulombic efficiency of 88.8% (Supplementary Fig. 28) when charged to 4.3 V. After 60 cycles, the capacity was 135 mAh g^−1^ with 81.8% retention (Fig. 5d, e). Good cycling stability of the 4.3 V and 4.4 V LCO SSLBs (Fig. 5f, g and Supplementary Fig. 29) at 0.2 C rate was also obtained, suggesting the utilization of this cage electrolyte can be extended to different types of cathodes.

In summary, we have developed an organic cage-based ionic conductor, Li-RCC1-ClO_4_, for the preparation of high-performance solid-state cathodes. The ionic cage structure not only contributes to the high ionic conductivity and ion transference number, while also offering solution-processing options, such as the slurry coating, for cathode preparation. As such, this approach may be more broadly transferable to other kinds of SSLBs. The organic cage catholyte is dissolved in the slurry and then recrystallizes and grow on the surface of the cathode particles during the coating process, building a continuous Li ion conducting network in the solid-state cathode. As a result, the SSLBs containing 20% of this cage type SSE in the solid-state cathodes (LiFePO_4_, NCM523, LCO) present small polarization and good cyclability at room temperature. Such molecular cage catholyte is fully compatible with current cathode manufacturing processes and have high potential for application in SSLBs. Future studies will focus on introducing additional advantages, such as better air/moisture stability and enhanced mechanical properties as well as ionic conductivity by using structured organic molecular additives such as organic cages.

## Methods

### Synthesis of RCC1 and RCC1-Cl

Ethylenediamine (520.0 mg, 8.65 mmol, 99.0%) was dissolved in methanol (212 mL) in a round-bottomed flask with ice bath. 1,3,5-Triformylbenzene (937.5 mg, 5.80 mmol, 98.0%) was dissolved in methanol (288 mL) and added slowly to the above ethylene diamine solution under nitrogen gas protection over 24 h. Sodium borohydride (765.0 mg, 20.15 mmol, 99.0%) was then added several times in small doses. The solution was stirred continuously during the reaction. After 12 h, 2.5 mL water was added to quench the reaction. The solvent was removed by a rotary evaporator after adding water. Subsequently, the resulting white powder was extracted with 50 mL chloroform twice. After that, the chloroform was removed under vacuum overnight and amine cage RCC1 was obtained as white powder (840 mg, ~80% yield)^42^. This was then purified using a Biotage Isolera four using a C18 reverse phase column with methanol/water as solvent. After removal of the solvent, RCC1 was obtained as a clear solid in a 42% overall yield (352 mg).

To prepare RCC1-Cl, RCC1 (500 mg, 0.612 mmol) was first added to chloroform (10 ml) with stirring. After the RCC1 had dissolved, hydrogen chloride (in dioxane, 2.3 ml, 9.18 mmol) was added dropwise to the solution. A large quantity of white precipitate appeared rapidly and the solution was stirred further for another 2 h. The resulting precipitate was collected by filtration then washed with chloroform (320 ml total) three times. RCC1-Cl was obtained as a white solid with a yield of 71% (532 mg) after being dried under vacuum at 90 °C^41^.

### Synthesis of RCC1-ClO_4_ and Li-RCC1-ClO_4_

The RCC1-Cl powder was dispersed in a 15 wt.% lithium perchlorate (99.0%)-ethyl acetate (100 mL, 99.9%) solution at 50 °C with stirring to allow ion exchange. The solution was changed with the fresh lithium perchlorate-ethyl acetate solution every 48 h three times. The solid product, RCC1-ClO_4_, was obtained with a yield of 60% by centrifugation and washed with ethyl acetate for three times. After ion exchange, the product was mixed with lithium perchlorate in ethanol-water solvent. The solvent was removed using a rotary evaporator. The final product, Li-RCC1-ClO_4_, was collected and dried at 80 °C in vacuum overnight.

### Structural characterization of Li-RCC1-ClO_4_ and the all-solid-state cathode with Li-RCC1-ClO_4_

Spectra for ^1^H nuclear magnetic resonance (NMR) analysis were obtained on a Bruker Advance 400 and 600 Spectrometer in D_2_O. Solution ^1^H NMR spectra were recorded at 300 MHz using a Bruker Avance 500 and ^13^C NMR spectra were recorded at 75 MHz. X-ray photoelectron spectroscopy (XPS) spectra were acquired by using Thermo scientific ESCALAB 250Xi with Al Kα-radiation. All reported binding energy values are calibrated to the graphitic C 1 *s* peak with a value of 284.5 eV. The samples were prevented from contacting with air through a vacuum transfer device which can transfer the samples into the analysis chamber of the XPS spectrometer without exposure to the air. Fourier transform infrared (FTIR) spectra were collected using a Thermoscientific Nicolet 6700 spectrometer. Raman measurements were recorded with the laser wavelength is 532 nm. Scanning electron microscope (SEM) images were gained with a FEI Quanta 400 FEG equipped with EDX (Apollo 40 SDD) operated at 10 kV. The focus-ion-beam-milled (FIB milling) and the corresponding SEM imaging of the cross-section samples were conducted in a dual-beam Nova 200 NanoLab UHRFEG system. XRD patterns were performed on a Bruker D8-advance X-ray diffractometer with Cu-Kα radiation. Thermogravimetry (TG) curves were obtained with a Seiko 6300 thermo-gravimetric analyzer under air flow with a heating rate of 10 °C min^−1^. Depth profiles of elemental distributions were obtained using time-of-flight secondary ion mass spectrometry (TOF-SIMS) (TOF.SIMS5-100). Bi^+^ ions at an accelerating voltage of 10 kV were used for the analysis and Cs^+^ was accelerated at 2 kV and 20 nA for sputtering. TOF-SIMS was used ex situ to probe the 3D distribution of the cathode components. For the non-in-situ tests, including FTIR, Raman, XRD, TG and TOF-SIMS, samples were placed on the sample stage and sealed in suitable stage container in an Ar-filled glovebox (O_2_ < 10 ppm, H_2_O < 1 ppm) until the tests begin, then open the sealed container and transfer sample stage for testing as soon as possible, and controls the ambient humidity below 20%.

### Electrochemical measurements and cells assembly

Ionic conductivity of the samples was measured by electrochemical impedance spectroscopy (EIS) using an EC-lab during the frequency range from 0.01 Hz to 7 MHz with alternating current amplitude of 10 mV. Samples with Au films grown on each side with thickness of ~25 µm and diameter of 8 mm by magnetron sputtering were sandwiched in CR2032 coin cells for tests. The ionic conductivity was calculated from Eq. (1):

1 $$\sigma =L/(R\cdot S)$$

where *R* is the bulk resistance, *L* and *S* are the thickness and area of the solid electrolyte, respectively, in which *S* is calculated by the contact area between the electrolyte and Au blocking electrode.

Linear sweep voltammetry (LSV) with a sweep rate of 1 mV s^−1^ between 0 and 6.0 V, was applied on cion cells with Au working electrode as a counter and Li foil as a reference electrode on each side of the testing electrolytes. The transference number was measured and calculated by alternating current (AC) impedance and direct-current (DC) polarization (with a DC voltage of 50 mV). Li-carbon composite was coated on both side of solid-state electrolyte as non-blocking electrodes (diameter of 8 mm) to make better contact between the solid-state electrolyte and electrodes than Li foil electrodes. The Li-carbon composite slurry, including Li-CNT composite, carbon black and styrene butadiene rubber (SBR), was mixed with a mass ratio of 80:10:10 in para-xylene. After stirring for 24 h, the slurry was then coated onto both sides of the electrolyte and dried at 80 °C under vacuum overnight^68^.

The Li transference number (*t*_Li+_) was calculated as in Eq. (2):

2 $${t}_{{{{{{{\rm{Li}}}}}}}^{+}}=\frac{{i}_{{{{{{\rm{s}}}}}}}\left(\Delta V{{\mbox{-}}}{i}_{{{{{{\rm{o}}}}}}}{R}_{{{{{{\rm{o}}}}}}}\right)}{{i}_{{{{{{\rm{o}}}}}}}\left(\Delta V{{\mbox{-}}}{i}_{{{{{{\rm{s}}}}}}}{R}_{{{{{{\rm{s}}}}}}}\right)}$$

where *I*_o_ is the initial current, *I*_s_ is the steady-state current, Δ*V* is the applied potential, *R*_o_ and *R*_s_ is the overall cell resistance value before and after polarization, respectively.

The electrochemical performance of the solid-state batteries was tested with a coin cell (CR2025) assembled in an argon-filled glove box. The lithium foil (99.9%, 400 µm, Tianjin Zhongneng Lithium Co. LTD) was used as anode in the cells and the P(IL-PEGDA) solid polymer (80~100 µm) electrolyte was used as solid electrolyte to ensure good interfacial contact. The P(IL-PEGDA) solid polymer electrolyte was prepared by mixing 1-Vinyl-3-butylimidazolium bis-(trifluoromethylsulfonyl)imide (Lanzhou Institute of Chemical Physics), poly(ethylene glycol) diacrylate (Mn = 1000 g mol^−1^, Aladdin) and LiTFSI (mass ratio = 28:5:12) first, then appropriate amount of anhydrous acetonitrile was added with stirring for 3 h. After that, phenylbis(2,4,6-trimethylbenzoyl)phosphine oxide as a photoinitiator (1 wt% of the monomers) was added in and stirred for 30 min. The mixture was blade-casted onto a glass substrate and photocured by a 365 nm ultraviolet light for 2 min, followed by vacuum-dried at 60 °C overnight to remove the anhydrous acetonitrile, and the P(IL-PEGDA) solid polymer electrolyte was obtained^56^. The cathode electrode slurry was prepared by adding LiFePO_4_ (KJGROUP), AB (Alfa Aesar), CNTs (2.4 wt% in N-methylpyrrolidone (NMP)), PVDF (Solef 5130) and Li-RCC1-ClO_4_ with a mass ratio of 71:4:2:3:20 into a mixture of NMP (99.9%, Aladdin) and methyl alcohol (anhydrous, ≥99.9%, Sigma-Aldrich) with a volume ratio of 1:1. The slurry was casted on Al foil (>99.3%, 16 μm, Hefei Kojing Material Technology Co., LTD) and dried at 80 °C for 12 h. To make intimate contact between the different components, the cathode electrode with LiFePO_4_ areal loading in the range from 1.0 to 4.0 mg cm^−2^ was roll pressed by a roller mill (MSK-HRP-01, Hefei Kojing Material Technology Co., LTD) to densities of 1.24~4.96 g cm^−3^. The weight ratio of the cathode components without the Li-RCC1-ClO_4_ additive is LiFePO_4_:AB:CNTs:PVDF = 71:4:2:3. While weight ratio of the cathode components with the PEO/LiClO_4_ additive is LiFePO_4_:AB:CNTs:PEO/LiClO_4_ = 71:4:2:23. For the battery coupled with the LLZO pellet (polished to ~300 µm before use), 5 µm P(IL-PEGDA) solid polymer electrolyte^56^ was in situ grown on one side of LLZO pellet facing the Li anode, while the cathode slurry was cast on another side of LLZO, then Al foil was covered on the sample before it dried at 80 °C. For the ionic conductivity tests of the solid-state cathodes, the cathode slurry only contains cage electrolyte and LiFePO_4_ at three different mass ratios (cage electrolyte:LiFePO_4_ = 10:90, 20:80, and 30:70) was casted on the Al foil with a ~25 µm Au layer; after it was dried and roll-pressed, ~25 µm Au film (diameter of 8 mm) was sputtered on another side of the cathode, then the symmetric cells were assembled in an argon-filled glove box for further tests. For the LCO and NCM523 cathodes, the weight ratio of the cathode components was LCO/NCM523: AB:CNTs:PVDF: Li-RCC1-ClO_4_ =74:2:1:3:20, the SSE between the cathode and anode can withstand high-voltage (>4.5 V) which is polymerized by 1-allyl-1-methyl- pyrrolidinium Bis (trifluoromethanesulfonyl) imide, 1H,1H,6H,6H-perfluorohexanediyl diacrylate, and vinyl ethylene carbonate. The Li|SPE|LiFePO_4_ (or LCO/NCM523) batteries were assembled layer by layer and sealed at 50 kg cm^−2^ by a battery sealing machine (MSK-110, Hefei Kojing Material Technology Co., LTD). The electrochemical performance of the batteries was measured on a Neware BTS battery tester in a room with air conditions to control the temperature at 25 ± 3 °C

## Supplementary information

Supplementary Information

## Data Availability

Additional data on methods, materials characterizations and electrochemical performance are available in Supplementary Information. Source data are provided with this paper.

## Source data

Source Data
