# Supplementary material for: Room temperature all-solid-state lithium batteries based on a soluble organic cage ionic conductor
Source: Nat Commun. 2022 Apr 19;13:2031. doi: 10.1038/s41467-022-29743-1 (PMC9018795; doi:10.1038/s41467-022-29743-1)
Supplement: Supplementary file 1 — Supplementary Information [file 41467_2022_29743_MOESM1_ESM.pdf]

Supplementary Information for
Room Temperature All-Solid-State Lithium Batteries Based on a Soluble Organic Cage Ionic Conductor

Jing Li[1,2,3], Jizhen Qi[2], Feng Jin[2], Fengrui Zhang[2], Lei Zheng[2], Lingfei Tang[2], Rong Huang[4], Jingjing Xu[2], Hongwei Chen[5], Ming Liu[6], Yejun Qiu[1*], Andrew I. Cooper[6*], Yanbin Shen[2*], and Liwei Chen[2,4,7*]

[1] Shenzhen Engineering Lab of Flexible Transparent Conductive Films, School of Materials Science and Engineering, Harbin Institute of Technology, Shenzhen, 518055, China

[2] i-Lab, Suzhou Institute of Nano-Tech and Nano-Bionics, Chinese Academy of Sciences, Suzhou 215123, China.

[3] School of Materials Science and Engineering, Suzhou University of Science and Technology, Suzhou 215009, China.

[4] Vacuum Interconnected Nanotech Workstation (Nano-X), Suzhou Institute of Nano-Tech and Nano-Bionics, Chinese Academy of Sciences, Suzhou 215123, China.

[5] College of Materials Science and Engineering, Huaqiao University, Xiamen 361021, P.R. China.

[6] Leverhulme Centre for Functional Materials Design, Materials Innovation Factory and Department of Chemistry, University of Liverpool, UK.

*[7] In-situ Center for Physical Sciences, School of Chemistry and Chemical Engineering, Shanghai Jiaotong University, Shanghai, 200240, China. *e-mail: yejunqiu@hit.edu.cn; aicooper@liverpool.ac.uk; ybshen2017@sinano.ac.cn; lwchen2008@sinano.ac.cn.*

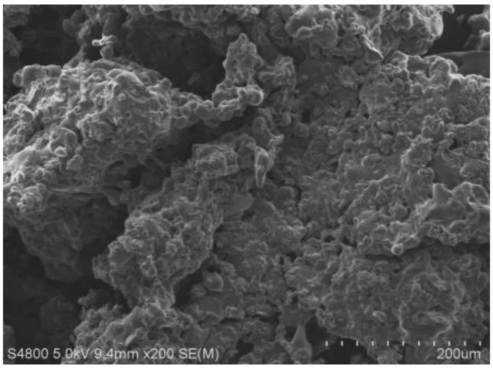

Supplementary Fig. 1. SEM image of the RCC1-Cl sample. The SEM image shows that the morphology of the RCC1-Cl sample is consistent with previous report[1].

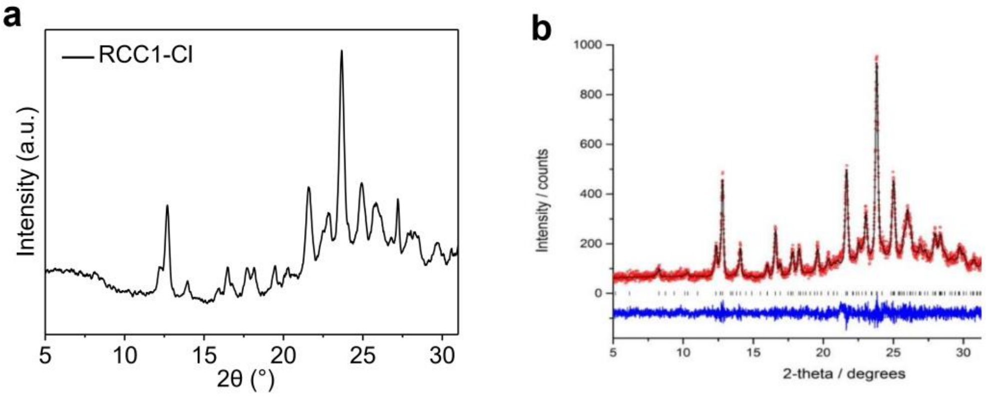

Supplementary Fig. 2. XRD pattern for RCC1-Cl. a in this work and **b** in literature[1].

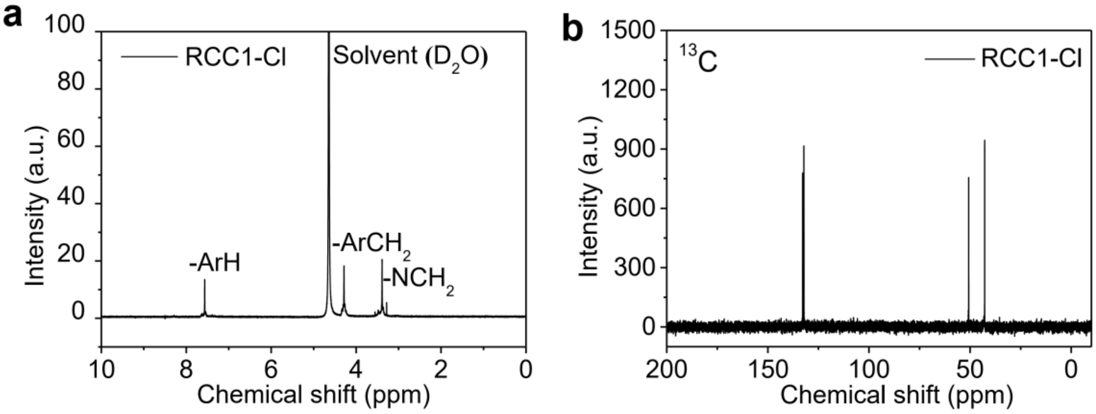

Supplementary Fig. 3. NMR spectra of RCC1-Cl. a ^{1}H and **b** ^{13}C spectra in D_2O. The chemical shifts of ^{1}H at 7.7, 4.4, 3.5 ppm and ^{13}C at 132.7, 132.1, 50.6, 42.8 are assigned to RCC1-Cl.

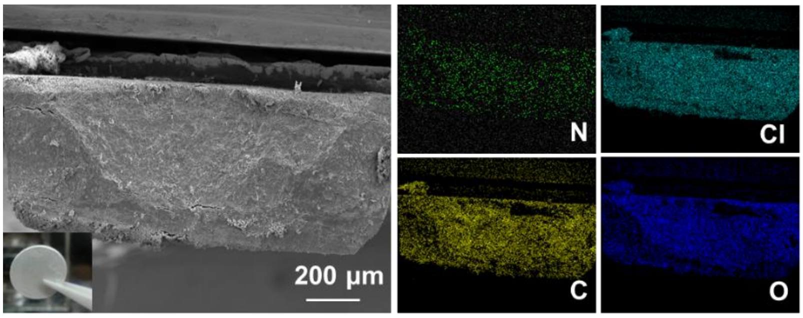

Supplementary Fig. 4. **SEM and EDX of the prepared Li-RCC1-ClO_4 solid pellet.** The dried Li-CON-TFSI powder was directly cold-pressed into pellets at room temperature. EDX analysis indicated that the major chemical components of framework (C, N, O) and corresponding ClO_4^- (Cl and O) are evenly distributed across the resulted pellet.

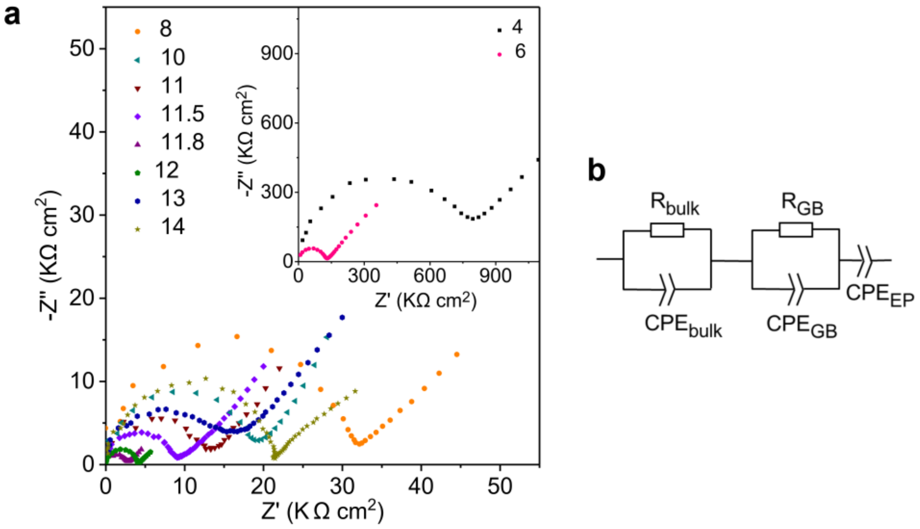

Supplementary Fig. 5. Impedance spectra and fitting model of different cells. a Electrochemical impedance spectra profiles of Li-RCC1-ClO_4 symmetrical Au blocking cells at different $[Li^+]/[-NH_2^+-]$ ratios. **b** Equivalent circuit used for fitting the EIS in Supplementary Fig. 5a. The impedance is normalized according to the thickness of electrolyte pellets. The equivalent circuit (RQ)(RQ)Q is used for the fitting (Q is a constant phase element, CPE, which represents an imperfect capacitor). The spectra were recorded in the frequency range from 7 MHz to 100 mHz. The resistance R_{bulk} and R_{GB} represent bulk and grain boundary resistance of the electrolyte, respectively. CPE_{EP} is the electrode polarization.

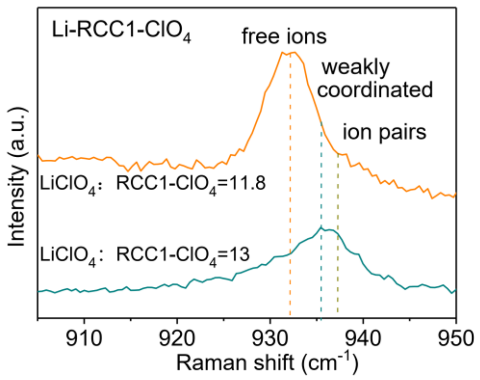

Supplementary Fig. 6. **Raman spectra of Li-RCC1-ClO_4 with different $LiClO_4$ content.** The Raman spectra showed that further increase in the content of the Li salt to a molar ratio of $[Li^+]/[-NH^{2+}-]$ from 11.8 (orange line) to 13 (green line) will result in an increase in residual undissociated $LiClO_4$.

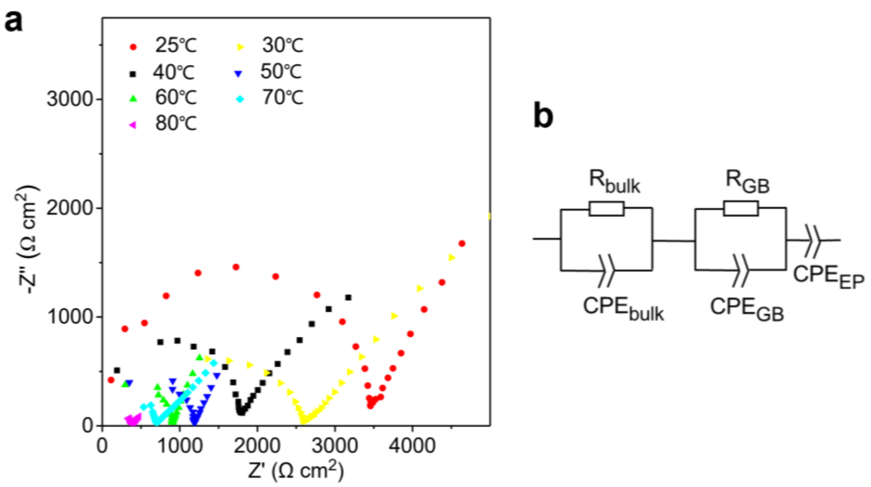

Supplementary Fig. 7. Impedance spectra and fitting model of Li-RCC1-ClO_4 at different temperatures. a Electrochemical impedance spectra profiles of Li-RCC1-ClO_4 symmetrical Au blocking cell at different temperatures. **b** Equivalent circuit used for fitting the EIS in Supplementary Fig. 7a. The pellet thickness for EIS measurements is 1160 µm. The equivalent circuit (RQ)(RQ)Q is used for the fitting (Q is a constant phase element, CPE, which represents an imperfect capacitor). The spectra were recorded in the frequency range from 7 MHz to 100 mHz. The resistance R_{bulk} and R_{GB} represent bulk and grain boundary resistance of the electrolyte, respectively. CPE_{EP} is the electrode polarization.

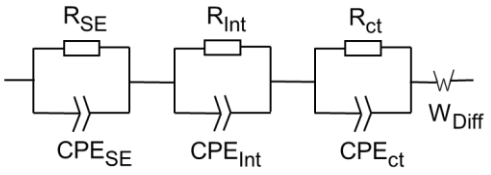

Supplementary Fig. 8. Equivalent circuit used to model impedance of symmetric Li-carbon composite|Li-RCC1-ClO_4|Li-carbon composite cells in Fig. 2d. The equivalent circuit (RQ)(RQ)(RQ)W is used for the fitting (Q is a constant phase element, CPE, which represents an imperfect capacitor, W assigns to the solid-state diffusion of Li^+ in Li-carbon composite electrodes.). The spectra were recorded in the frequency range from 7 MHz to 100 mHz. The resistance R_{SE}, R_{Int} and R_{ct} represent resistance of electrolyte, electrolyte/Li-carbon composite interface and charge transfer, respectively.

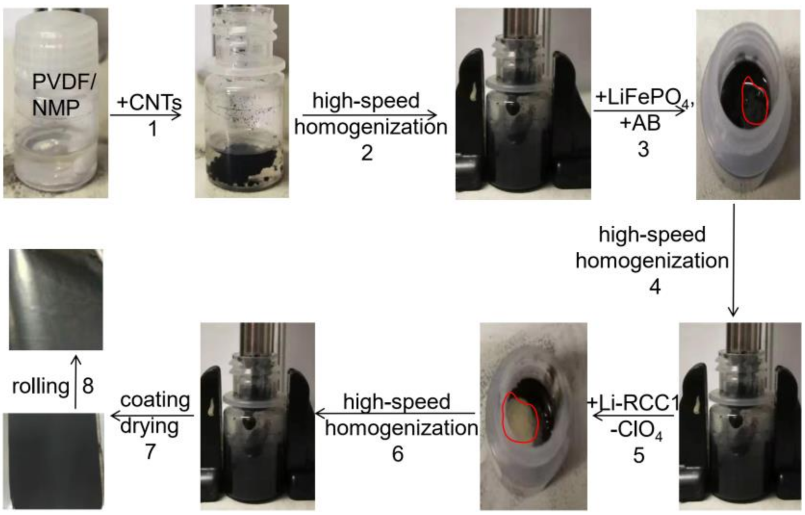

Supplementary Fig. 9. Processing of the solid-state cathode. The slurry was mixed and homogenized at high-speed to make components of the cathode distributed evenly, then the slurry was spread on the aluminum foil and rolled after drying. It was considered that intimate contact between the different components inside the cathode can enable enhanced ionic and electronic conductivity; thus, the cathode electrodes with original thickness of 50–60 μm (contain 17 μm Al foil) were pressed to 25–30 μm.

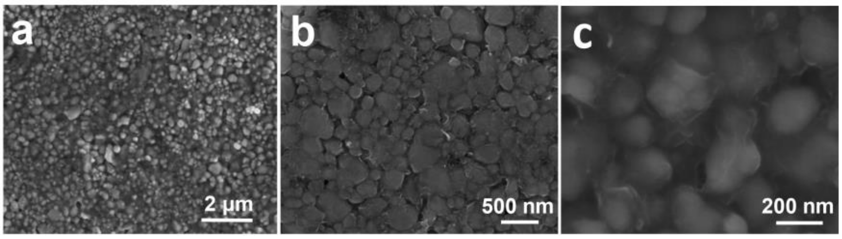

Supplementary Fig. 10. SEM images of the roll pressed solid state cathode with Li-RCC1-ClO_4 at different magnifications. a at low magnification, **b** at medium magnification, and **c** at high magnification. It can be concluded that the ionic Li-RCC1-ClO_4 conductor and the electronic AB/CNTs conductor are dispersed around $LiFePO_4$ particles, forming both continuous ion and electron conducting pathways for Li ions and electron migration, respectively.

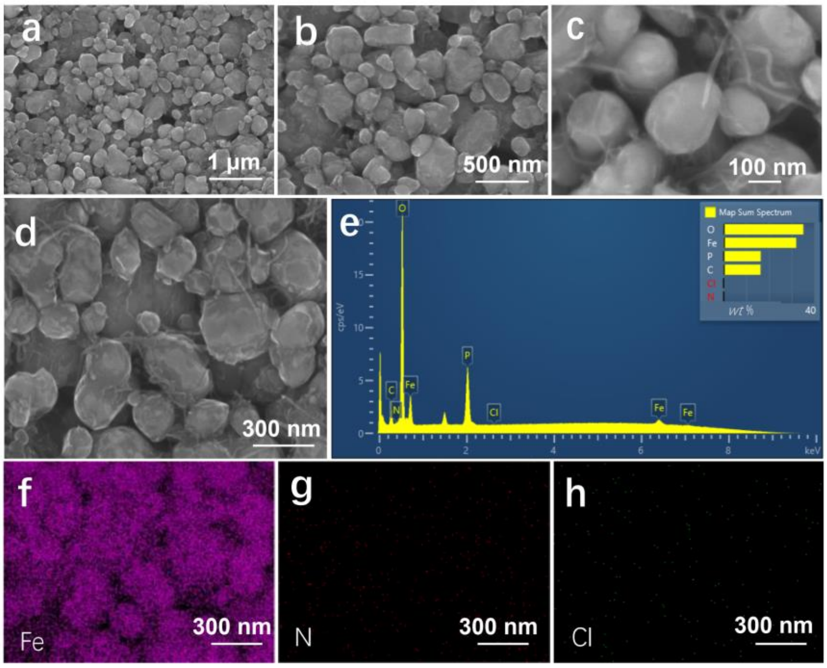

Supplementary Fig. 11. SEM and EDS of the cathode without Li-RCC1-ClO_4 catholyte. **a–c** SEM images of the roll pressed solid state cathode without Li-RCC1-ClO_4 at different magnification. **d** EDS image and **e** EDS element types of the cathode, **f–h** elemental maps of Fe, N and Cl, respectively.

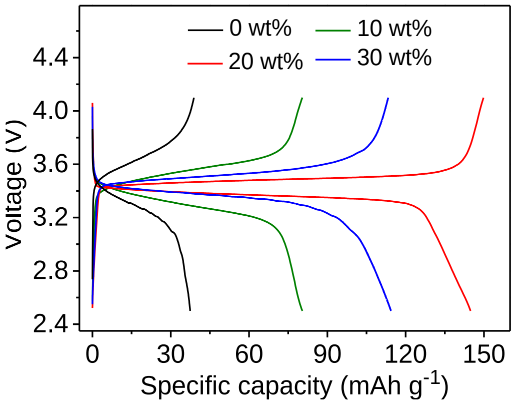

Supplementary Fig. 12. Optimization of the Li||$LiFePO_4$ cell formulation composition with Li-RCC1-$LiClO_4$ catholyte. The cells were tested at 0.1 C under room temperature.

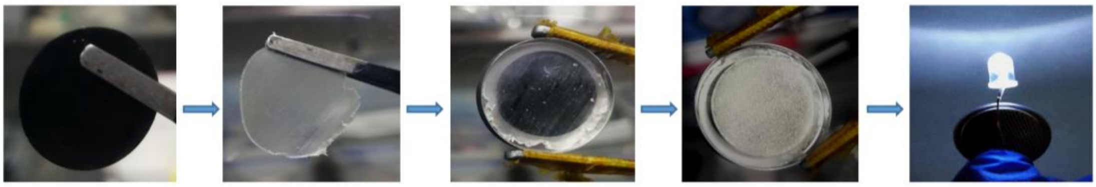

Supplementary Fig. 13. Assembly of the Li||$LiFePO_4$ all-solid-state battery. The Li||$LiFePO_4$ cell was assembled by placing a polymer solid electrolyte P(IL-PEGDA) in

between solid-state cathode and a lithium foil anode.

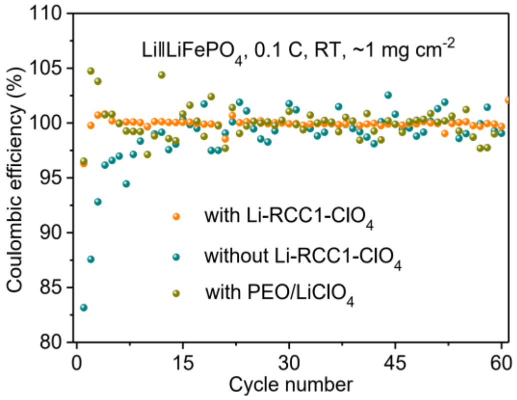

Supplementary Fig. 14. Coulombic efficiency of the different SSLBs in Fig. 4c. The cells using $LiFePO_4$ solid-state cathodes (loading: ~1.0 mg cm^{-2}) with and without Li-RCC1-ClO_4, as well as with PEO/$LiClO_4$ polymer as the catholyte were tested at room temperature.

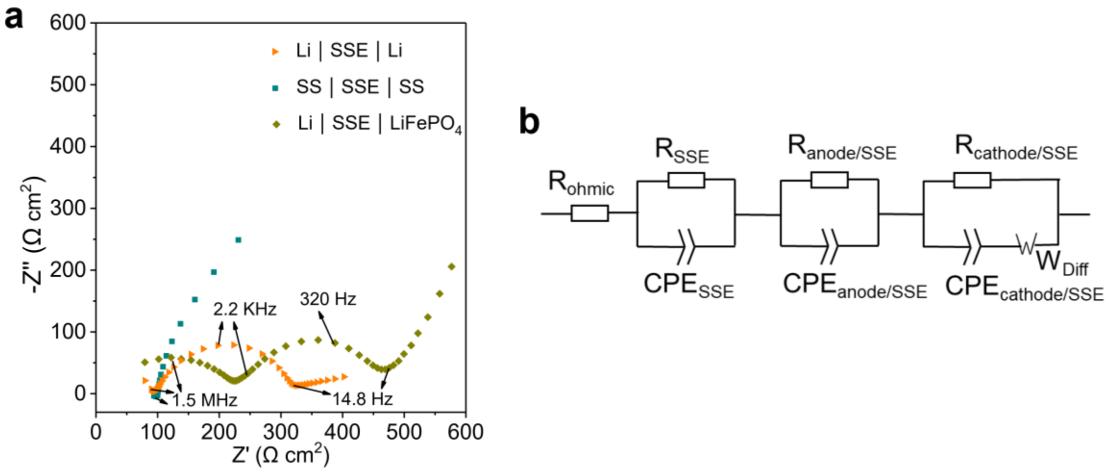

Supplementary Fig. 15. Impedance spectra and fitting model of different cells. a Electrochemical impedance spectra profiles of Li|SSE|Li, SS|SSE|SS and Li|SSE|$LiFePO_4$ cells. **b** Equivalent circuit used for fitting the EIS of Li|SSE|$LiFePO_4$ cells in Fig. 4d. In the EIS curve of Li|SPE|Li symmetry cell in Supplementary Fig. 15a, two semicircles can be clearly observed: one at the high frequencies (>1.5 MHz) is assigned as the resistance of the SSE, which is ~100 Ω cm^2, and the other at middle and low frequencies is assigned to the SSE/Li interface resistance. The area specific resistance (ASR) of SSE/Li interface is ~110 Ω cm^2. The equivalent circuit R(RQ)(RQ)(R(QW)) is used for fitting the EIS of Li|SSE|$LiFePO_4$ in Fig. 4d (Q is a constant phase element, CPE, which represents an imperfect capacitor, W assigns to the solid-state diffusion of Li^+ in cathode electrodes.). Wherein R_{ohnic} corresponds to the ohmic resistance of the current collectors and cell connections, R_{SE} represents the resistance of the solid electrolyte, and $R_{anode/SSE}$ and $R_{cathode/SSE}$ are associated with the interfacial charge transfer occurring at the anode's and cathode's surface, respectively. W_{Diff} is assigned

to the solid-state diffusion of Li^+ in cathode electrode.

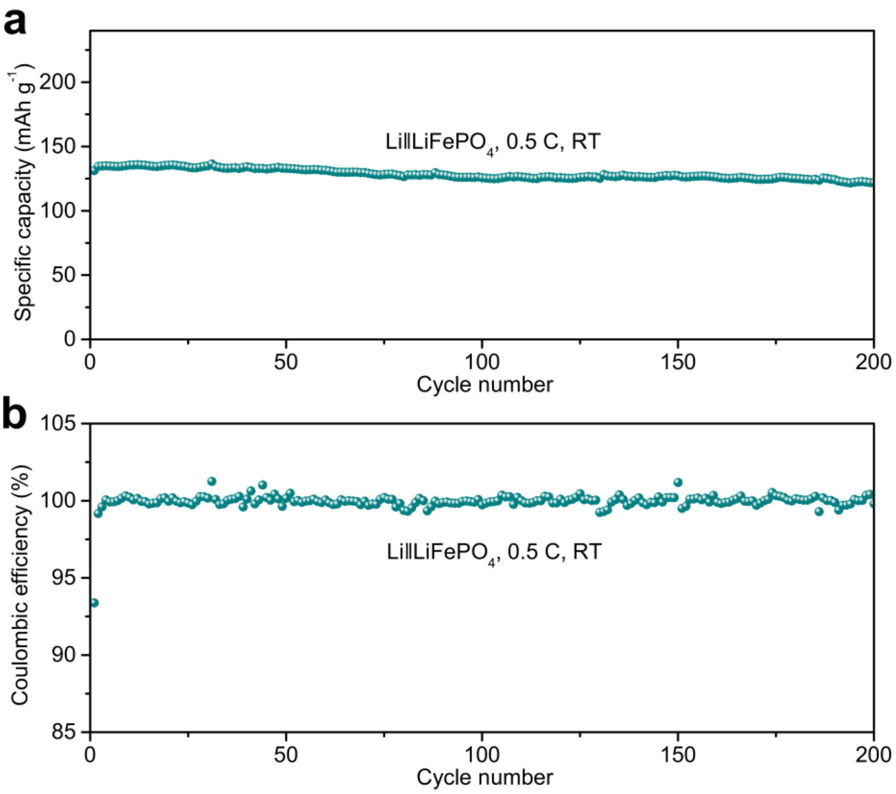

Supplementary Fig. 16. Electrochemical performance of the all-solid-state battery with Li-RCC1-ClO_4 catholyte. a Cycling performance and **b** Coulombic efficiency of the Li||$LiFePO_4$ all-solid-state cell with Li-RCC1-ClO_4 at 0.5 C under room temperature. ($LiFePO_4$ loading: 1.0 mg cm^{-2}).

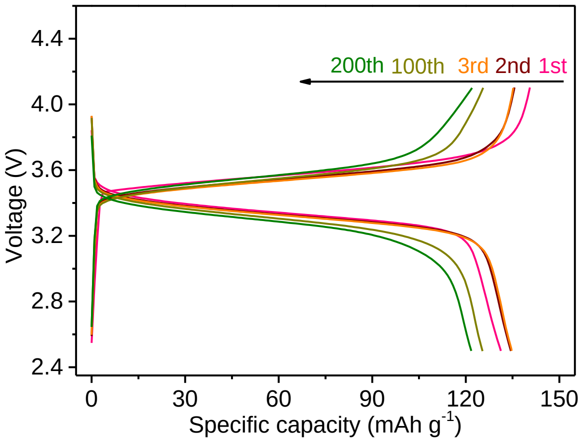

Supplementary Fig. 17. Voltage-capacity curves for the Li||$LiFePO_4$ cell with Li-RCC1-ClO_4 catholyte. The cell was cycled at 0.5 C under room temperature with $LiFePO_4$ loading of 1.0 mg cm^{-2}.

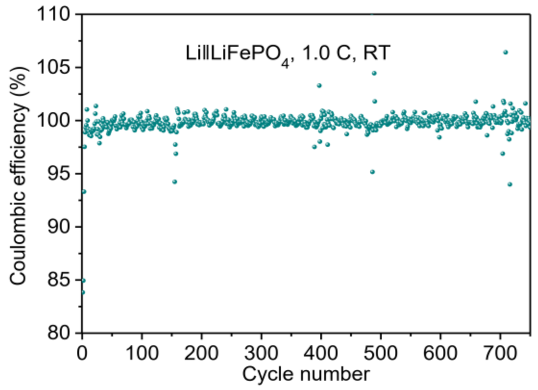

Supplementary Fig. 18. Coulombic efficiency of the all-solid-state cell with Li-RCC1-ClO_4 catholyte in Fig. 5a. The cell was cycled at 1.0 C under room temperature with $LiFePO_4$ loading of about 1.0 mg cm^{-2}.

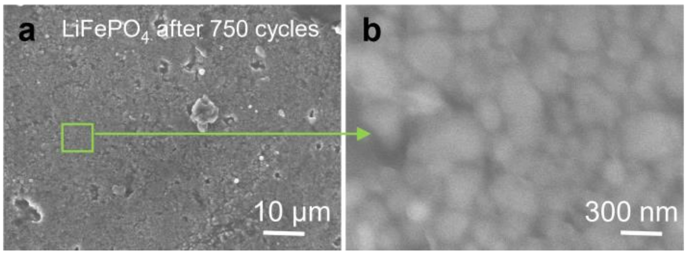

Supplementary Fig. 19. SEM on the cycled $LiFePO_4$ cathode of the all-solid-state Li|| $LiFePO_4$ cell after 750 cycles at 1 C rate. a at low magnification, **b** at high magnification.

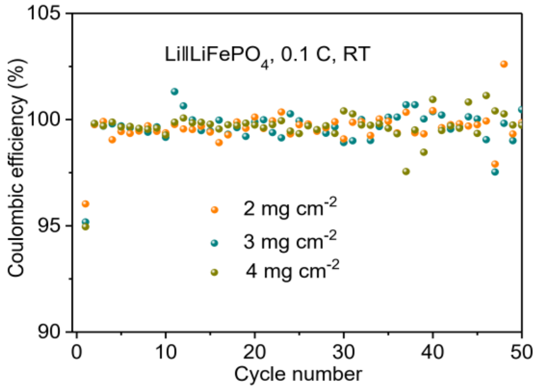

Supplementary Fig. 20. Coulombic efficiency of the all-solid-state cell with Li-RCC1-ClO_4 catholyte in Fig. 5b. The cells were cycled at 1.0 C under room temperature with different $LiFePO_4$ loadings.

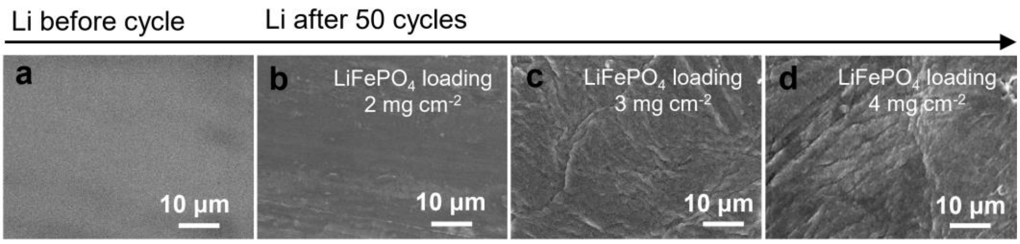

Supplementary Fig. 21. SEM on the Li anode of the all-solid-state Li||$LiFePO_4$ cells. a Li anode before cycling, **b-d** Li anode after cycling with $LiFePO_4$ areal loading of 2, 3 and 4 mg cm^{-2}, respectively.

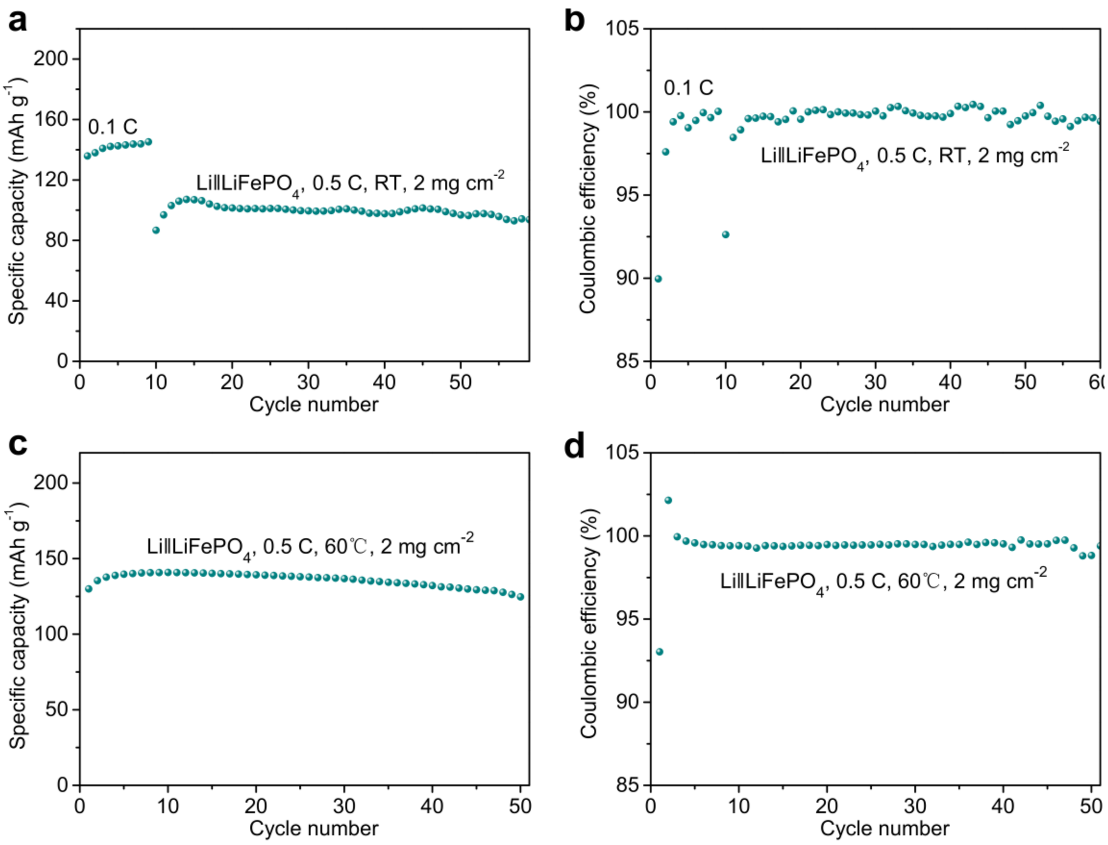

Supplementary Fig. 22. Electrochemical performance of the all-solid-state battery with Li-RCC1-ClO_4 catholyte at different temperatures. a, **c** Cycling performance and **b**, **d** Coulombic efficiency of the Li||$LiFePO_4$ all-solid-state cell with Li-RCC1-ClO_4 at 0.5 C under different temperature. **a**, **b** room temperature. **c**, **d** 60 °C.

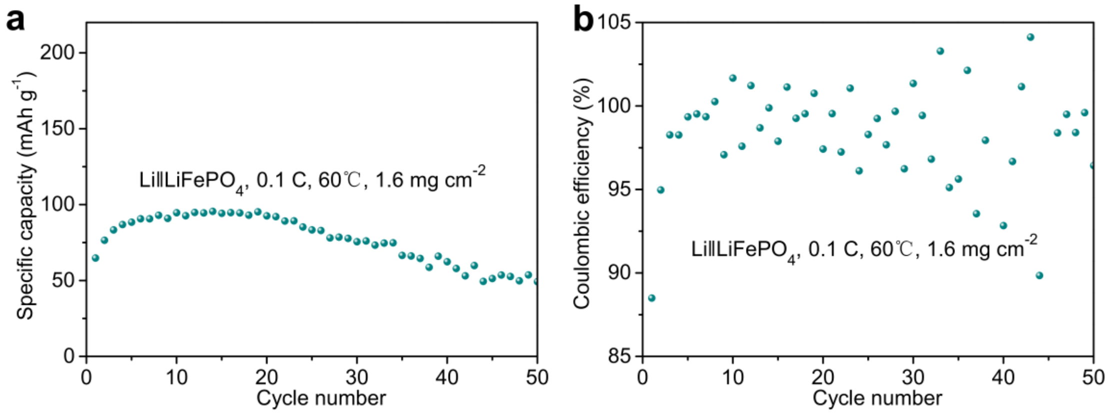

Supplementary Fig. 23. Electrochemical performance of the all-solid-state battery with PEO/$LiClO_4$ catholyte. a Cycling performance and **b** Coulombic efficiency of the Li||$LiFePO_4$ solid-state cell with PEO/$LiClO_4$ at 0.1 C at 60 °C.

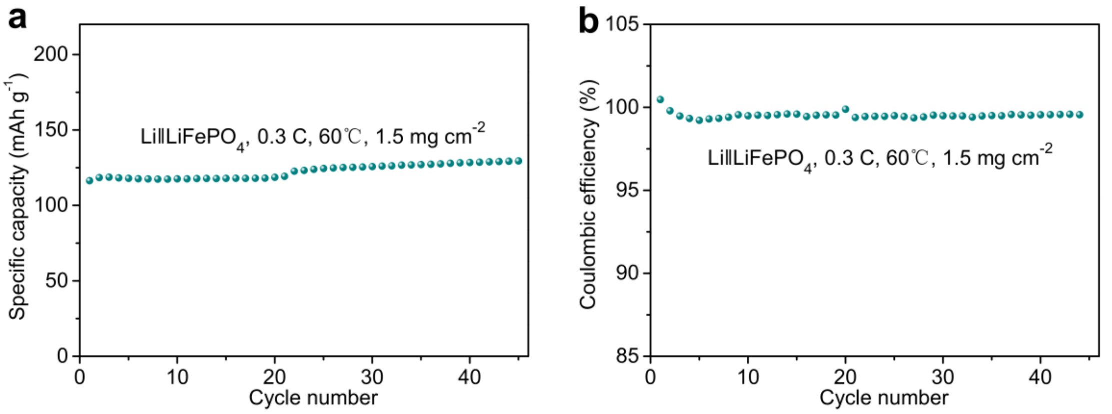

Supplementary Fig. 24. Electrochemical performance of the all-solid-state battery with Li-RCC1-ClO_4 catholyte. a Cycling performance and **b** Coulombic efficiency of the Li||$LiFePO_4$ all-solid-state cell with Li-RCC1-ClO_4 at 0.3 C under 60 °C.

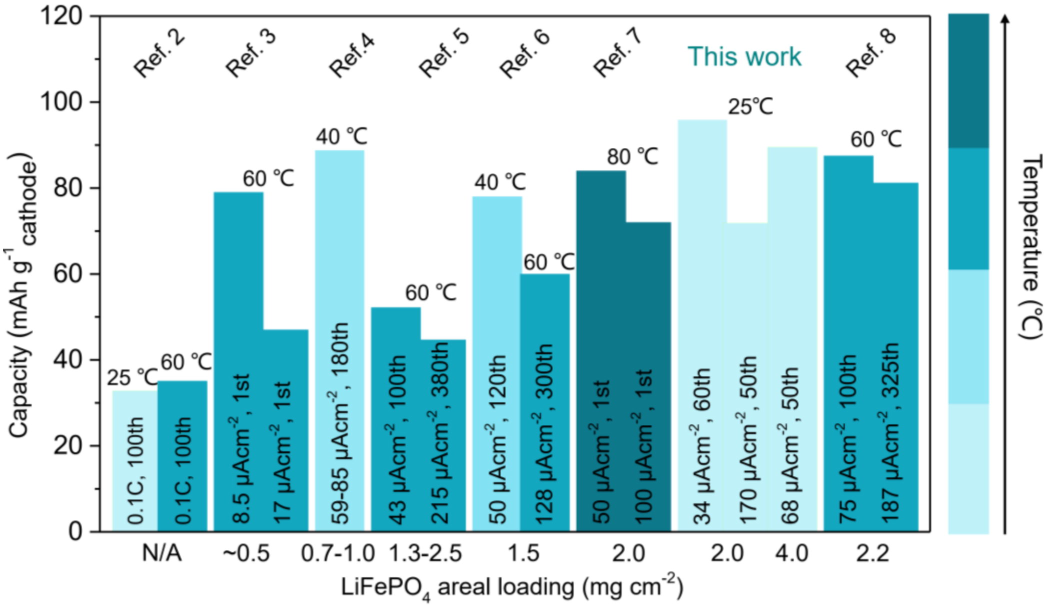

Supplementary Fig. 25. Performance comparison for different $LiFePO_4$ based solid batteries. The details are provided in the Supplementary Table 3.

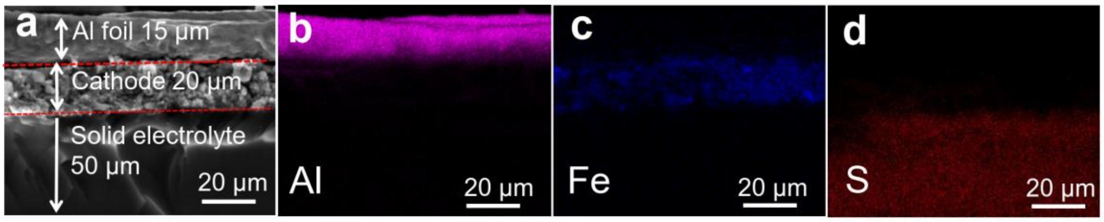

Supplementary Fig. 26. Cross-sectional SEM and EDS of the cycled Li||$LiFePO_4$ all-solid-state cell. a SEM image of the cycled solid-state cell after 50 cycles. **b–d** elemental maps of Al, Fe and S, respectively.

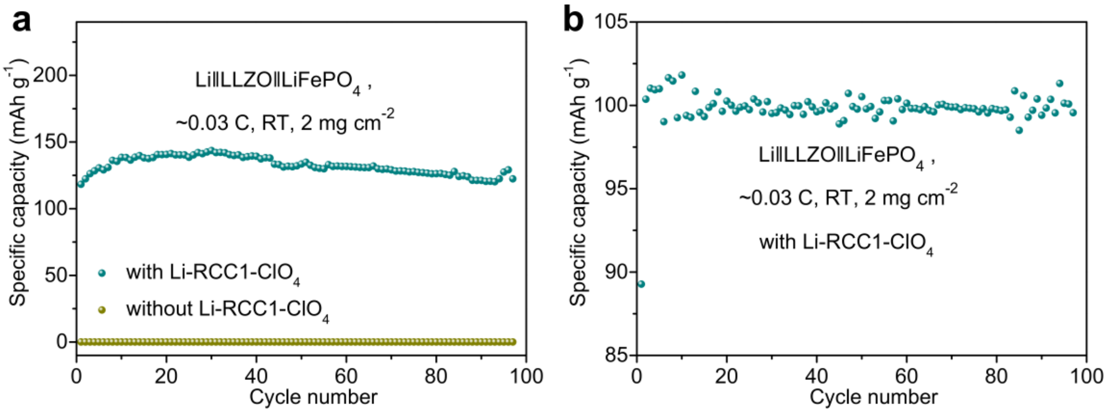

Supplementary Fig. 27. Electrochemical performance of the all-solid-state batteries with Li-RCC1-ClO_4 catholyte and LLZO solid state electrolyte. a Cycle performance and **b** Coulombic efficiency at 0.03 C.

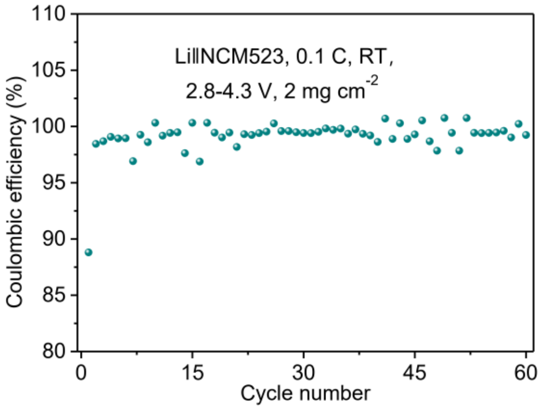

Supplementary Fig. 28. Coulombic efficiency corresponding to the cycling of Li||NCM523 all-solid-state cells showed in Fig. 5d. The cell was cycled during 2.8-4.3 V at 0.1 C under room temperature.

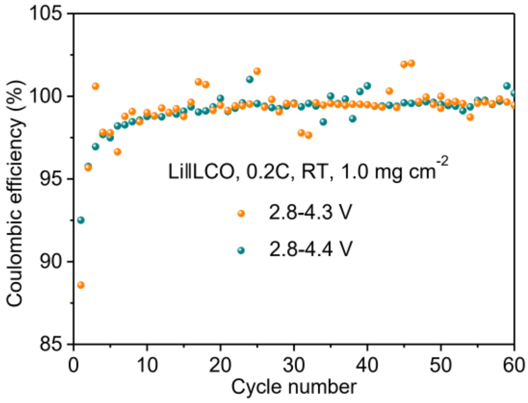

Supplementary Fig. 29. Coulombic efficiency corresponding to the cycling of Li||LCO all-solid-state cells showed in Fig. 5f. The cells were cycled during 2.8-4.3 V and 2.8-4.4 V at 0.2 C under room temperature.

Supplementary Table 1 Ionic conductivity (10^{-5} S cm^{-1}) of the solid cathode with different Li-RCC1-ClO_4 content and $LiFePO_4$ areal loading.

LFP areal loading (mg cm^{-2})	1	2	3	4	5	6
10% SSE	0.75	1.53	0.94	2.83	1.89	2.71
20% SSE	5.53	4.82	4.00	5.78	4.11	3.60
30% SSE	4.59	4.01	6.09	5.46	5.07	5.16

Supplementary Table 2 Summary of fitted data for the different cells. X^2 is the square of the standard deviation between the original and the calculated spectrum.

Battery configuration	R_{SSE} (Ω cm^2)	CPE_{SSE} [nF s(a1-1)]	R_{anode} (Ω cm^2)	CPE_{Anode} [nF s(a2-1)]	$R_{cathode}$ (Ω cm^2)	$CPE_{cathode}$ [nF s(a3-1)]	X^2
Li|SSE|Li $FePO_4$ (with Li-RCC1-ClO_4)	103	9.4	110	1680	255	2210	0.020
Li|SSE|$LiFePO_4$ (without Li-RCC1-ClO_4)	95	22.4	112	5510	785	2045	0.014
Li|SSE|$LiFePO_4$ (with PEO)	106	23.5	111	7120-	1520	5880	0.038
SS|SSE|SS	100	2.0-	-	-	-	-	0.015
Li|SSE|Li	96	1.5	112	6664	-	-	0.017

Supplementary Table 3 Electrochemical Performance of $LiFePO_4$-based Solid Batteries.

Reference	Cathode	Areal density (mg/cm^2)	Electrolyte	Temperature (℃)	Voltage Range (V)	Capacity ($mAh/g_{cathode}$)	Rate (Max.)
2	LiFePO4:LLZO: Super P:PVDF (24:56:10:10)	N/A	LLZO/LiTFSI/ polymer (45:45:10)	RT/60	2.0-4.2 V	32.8 mAh/g (0.1 C*100th), RT	35.1 mAh/g (0.1 C*100th), 60 ℃
3	LiFePO4:AB :PEO-LiTFSI (5:3:2)	~0.5	PEO /BPEG/LATP	60	2.5-3.9 V	79 mAh/g (0.1 C*1st)	47 mAh/g (2 C*1st)
4	LiFePO4: Super P: PEO-LiTFSI-LVC (6:1:3)	0.7-1	PEO-LiTFSI-LVC	40	2.5-3.9 V	88.8 mAh/g (0.5 C*180th)	~
5	LiFePO4:UIO/Li-IL :Ketjen black (4:4:2)	1.3-2.5	UIO/Li-IL	60	2.8-4.0 V	52.2 mAh/g (0.2 C*100th)	44.7 mAh/g (1 C*380th)
6	LiFePO4:PEO-$LiClO_4$:AB(60:25:15)	1.5	PI/PEO/ LiTFSI	40, 60	2.5-3.8 V	78 mAh/g (0.2 C*120th), 40 ℃	60 mAh/g (0.5 C*300th), 60 ℃
7	LiFePO4:AB :cross-linked PEO: LiTFSI (60:12:20:8)	2	$LiZr_2(PO_4)_3$	80	2.7-3.8 V	84 mAh/g (50 $\mu A\cdot cm^{-2}$*1st)	72 mAh/g (100 $\mu A\cdot cm^{-2}$*1st)
8	LiFePO4:PEO -$LiClO_4$:Super P (7:2:1)	2.2	PEO-LiTFSI/Li_3PS_4	60	2.8-3.8 V	87.5 mAh/g (0.2 C*100th)	81.2 mAh/g (0.5 C*325th)
Our work	LiFePO4:Li-RCC1-ClO_4: PVDF:AB+CNTs (74:20:3:3)	2, 4	P(IL-PEGDA) SPE	25	2.5-4.1 V	96.9 mAh/g (0.1 C*60th), 91.6 mAh/g (0.1 C*50th)	71.8 mAh/g (0.5 C*50th)

Supplementary References

1. Liu, M. *et al.* Three-dimensional protonic conductivity in porous organic cage solids. *Nat. Commun.,* **7** (2016).
2. Yan, X., Li, Z., Wen, Z. & Han, W. $Li/Li_7La_3Zr_2O_{12}/LiFePO_4$ all-solid-state battery with ultrathin nanoscale solid electrolyte. *J. Phys. Chem. C* **121**, 1431-1435 (2017).
3. Yang, L. et al. Flexible composite solid electrolyte facilitating highly stable "soft contacting" Li-electrolyte interface for solid state lithium-ion batteries. *Adv. Energy Mater.* **7**, 1701437 (2017).
4. Yuan, X. F. *et al.* A polyoxometalate-based polymer electrolyte with an improved electrode interface and ion conductivity for high-safety all-solid-state batteries. *J. Mater. Chem. A* **7**, 15924 (2019).
5. Wu, J. F. & Guo, X. Nanostructured metal–organic framework (MOF)-derived solid electrolytes realizing fast lithium ion transportation kinetics in solid-state batteries. *Small* **15**, 1804413 (2019).
6. Wan, J. *et al.* Ultrathin, flexible, solid polymer composite electrolyte enabled with aligned nanoporous host for lithium batteries. *Nat. Nanotechnol.* **14**, 705-711 (2019).
7. Li, Y. *et al.* Mastering the interface for advanced all-solid-state lithium rechargeable batteries. *PNAS.* **22**, 13313-13317 (2016).
8. Chen, S. *et al.* In-situ preparation of poly(ethylene oxide)/Li_3PS_4 hybrid polymer electrolyte with good nanofiller distribution for rechargeable solid-state lithium batteries. *J. Power Sources* **387**, 72-80 (2018).
